# An Efficient Compressive Sensed Video Codec with Inter-Frame Decoding and Low-Complexity Intra-Frame Encoding

**DOI:** 10.3390/s23031368

**Published:** 2023-01-26

**Authors:** Evgeny Belyaev

**Affiliations:** Information Technologies and Programming Faculty, ITMO University, Kronverksky Pr. 49, bldg. A, St. Petersburg 197101, Russia; eabelyaev@itmo.ru

**Keywords:** fast video encoding, compressive sensing

## Abstract

This paper is dedicated to video coding based on a compressive sensing (CS) framework. In CS, it is assumed that if a video sequence is sparse in some transform domain, then it could be reconstructed from a much lower number of samples (called measurements) than the Nyquist–Shannon theorem requires. Here, the performance of such a codec depends on how the measurements are acquired (or sensed) and compressed and how the video is reconstructed from the decoded measurements. Here, such a codec potentially could provide significantly faster encoding compared with traditional block-based intra-frame encoding via Motion JPEG (MJPEG), H.264/AVC or H.265/HEVC standards. However, existing video codecs based on CS are inferior to the traditional codecs in rate distortion performance, which makes them useless in practical scenarios. In this paper, we present a video codec based on CS called CS-JPEG. To the author’s knowledge, CS-JPEG is the first codec based on CS, combining fast encoding and high rate distortion results. Our performance evaluation shows that, compared with the optimized software implementations of MJPEG, H.264/AVC, and H.265/HEVC, the proposed CS-JPEG encoding is 2.2, 1.9, and 30.5 times faster, providing 2.33, 0.79, and 1.45 dB improvements in the peak signal-to-noise ratio, respectively. Therefore, it could be more attractive for video applications having critical limitations in computational resources or a battery lifetime of an upstreaming device.

## 1. Introduction

In recent years, more and more applications where a video upstreaming device is limited by its resources, such as the battery lifetime and the platform performance, have been developed. Examples include deep space missions [1], wireless endoscopy [2], wireless multimedia sensors [3,4], the Internet of Underwater Things [5], and high-speed video capturing [6]. The basic solution for these applications is traditional block-based intra-frame video coding, such as x264 [7], which is an open-source implementation of video encoding according to the H.264/AVC [8] standard. Video encoding through x264 includes several complexity profiles from *veryslow* to *ultrafast*. The *veryslow* profile corresponds to the maximum possible optimization of the codec parameters such that the compression performance is the highest while the encoding speed is the lowest. In contrast, the *ultrafast* profile corresponds to the maximum possible encoding speed. In [9], it was shown that x264 provides close to optimum rate distortion performance for the H.264/AVC standard when computational complexity is significantly restricted. The main goal of this paper is to answer the following question: Is it possible to compress faster than the traditional block-based intra-frame video coding, such as with x264 in an *ultrafast* profile, while having better compression performance?

One of the promising video coding concepts that can potentially provide lower encoding complexity is distributed video coding (DVC), where the coding complexity is shifted from the encoder to the decoder [10]. There are two main DVC implementations which are still under development:

1. DVC based on syndrome coding [11,12] or overlapped arithmetic coding [13];
2. DVC based on compressive sensing (CS) [3].

In the first implementation (e.g., DISCOVER [11]), some frames (called *key* frames) are intra-encoded via conventional video coding based on H.264/AVC in Intra mode. The remaining frames (called *Wyner–Ziv* (WZ) frames) are divided into non-overlapped blocks for which a 2D discrete cosine transform (DCT) is performed. The resulting DCT coefficients are converted into several binary vectors. Then, a syndrome of an error-correcting code is computed for each vector and transmitted to the decoder. At the decoder side, already-reconstructed frames are used in order to obtain the interpolated version of the decoded frame (called *side information*). Then, the received syndromes and the side information are used for syndrome decoding, considering the binary vectors extracted from the side information as a sum of the vectors obtained at the encoder and some errors in a *virtual channel*. Several works [11,12,13] reported that this approach outperforms H.264/AVC (Intra) in compression performance. However, the utilization of H.264/AVC makes the whole architecture more sophisticated, since it requires having two different encoders at the same upstreaming device. Moreover, since WZ frame encoding includes block-based DCT, binarization, and syndrome coding, it is very unlikely that it would be significantly less complex than the frame encoding via existing fast software implementations of H.264/AVC (Intra), such as via the x264 [7] encoder in an *ultrafast* profile.

This paper is dedicated to DVC based on CS, which potentially could provide lower encoding complexity compared with x264 in an *ultrafast* profile. In CS, it is assumed that if a video sequence is sparse in some transform domain, then it could be reconstructed from a much lower number of samples (called measurements) than the Nyquist–Shannon theorem requires [14,15]. The measurements could be quantized and entropy-encoded. As a result, the complexity and rate distortion (RD) performance of such a codec depend on how the measurements are acquired, quantized, and compressed and how the video is reconstructed from the decoded measurements. In [16], we proposed the CS-JPEG video codec, which outperformed other codecs based on CS such as DISCOS [17], DCVS [18], MH-BCS [19], and MC-BCS [20]. However, CS-JPEG from [16] has the following drawbacks. First, in [16], a video was recovered via the iterative shrinkage-thresholding Algorithm (ISTA), where VBM3D was used as the thresholding operator. Since VBM3D was originally developed for video denoising, it performs a lot of redundant operations at each iteration of ISTA, such as hard thresholding (which is used in order to obtain an initial estimation of a video signal before the empirical Wiener shrinkage) and block matching (which does not use motion vectors obtained at previous iterations). As a result, the decoding speed in [16] is too slow, even for low-frame resolutions. In [21], a fast thresholding algorithm was proposed. However, it does not take into account the motion between neighbor frames. Therefore, there is still room for the ISTA improving from both the complexity and performance points of view. Second, the encoders in [16,21] as well as in DISCOS, DCVS, MH-BCS, and MC-BCS allow only setting the number of measurements and corresponding quantization levels for all frames. As a result, the output bit rate depends on the statistical properties of an input video sequence. However, in order to fit the channel capacity, the upstreaming applications should have a rate control algorithm which provides a target bit rate by adaptive selection of the number of measurements and the quantization level for each frame. Therefore, the rate control for the CS-JPEG codec is needed.

In this paper, we address the issues described above and evaluate the performance of the resulting video codec. The main contributions of this paper are the following:

1. We introduce an accurate rate control algorithm based on packet dropping which does not lead to a noticeable increase in encoding complexity.
2. We propose fast randomized thresholding for the ISTA, which pseudo-randomly selects the shrinkage parameters at each iteration and shows that compared with the ISTA with VBM3D [22,23], it is significantly less complex and provides better recovery performance.
3. We show detailed comparisons of the proposed CS-JPEG codec with conventional optimized competitors, such as MJPEG, x264 (Intra), and x265 (Intra) [24] (fast software implementation of H.265/HEVC [25]) in *ultrafast* profiles. The comparisons demonstrate that the CS-JPEG codec provides much faster encoding and more accurate rate control. Moreover, it shows better compression performance for video sequences with low and medium motion levels.

The rest of this paper is organized as follows. Section 2 overviews the work related to the CS-JPEG codec which was previously performed by the authors in [16,21,23]. Section 3 describes the proposed rate control. Section 4 introduces the proposed fast randomized thresholding for the ISTA. Section 5 provides a comparison with the conventional intra codecs. Section 6 concludes the presented results.

## 2. Overview of CS-JPEG Video Codec Based on Compressive Sensing

### 2.1. Research Problem Statement

The CS task for a video sequence can be formulated in the following way. Let us define a frame $X_{i}\in R^{W\times H}$ with an index *i* as a 2D signal of a size W×H pixels (We use the following notation. The column vectors and matrices are denoted by boldfaced lowercase and uppercase letters, respectively, (e.g., v and A), and vec(A) concatenates columns of A into a vector). First, linear measurements for $X_{i}$ are acquired as $y_{i}=\Phi_{i}x_{i}$, where $x_{i}=$vec$\left(X_{i}\right)$, $\Phi_{i}\in R^{M_{i}\times WH}$ and $M_{i}<WH$ denotes the measurement (or sensing) matrix for the *i*th frame. As was shown in [23], the task of reconstruction of the frame $x_{i}$ from measurements $y_{i}$ can be efficiently solved with the ISTA, where the frames at iteration *k* are estimated as

(1) $$\left[{\hat{x}}_{1}^{k},\cdots ,{\hat{x}}_{F}^{k}\right]=\mathrm{soft}\left(\left[{\tilde{x}}_{1}^{k},\cdots ,{\tilde{x}}_{F}^{k}\right],\sigma_{k}\right),$$

where soft(.) is a soft thresholding operator with a threshold

(2) $$\sigma_{k}=\sigma_{0}\left(1-{\left(k/K\right)}^{2}\right),$$

where $\sigma_{0}$ is an initial threshold, *K* is the number of iterations, and

(3) $${\tilde{x}}_{i}^{k}={\hat{x}}_{i}^{k-1}+\beta \Phi_{i}^{T}\left(y_{i}-\Phi_{i}{\hat{x}}_{i}^{k-1}\right),$$

where 0<β<2 is the step size [23] (usually β=1.75).

In order to provide an efficient video coding based on the CS framework, we should answer the following questions:

1. Which measurement matrix $\Phi_{i}$ is the most efficient?
2. How do we efficiently transmit $\Phi_{i}$ to the decoder?
3. How do we quantize and compress the measurements $y_{i}$?
4. How do we choose the best combination of the quantization level and the sensing rate $R_{i}=\frac{M_{i}}{WH}$?
5. How do we choose the soft thresholding operator soft(.)?
6. How do we choose the best combination of parameters $\sigma_{0},K$ for the ISTA?

The rest of this section is dedicated to these questions.

### 2.2. Measurement (or Sensing) Matrix Selection and Quantization

In several video CS works [26,27], the block-based random measurements based on a Gaussian random projection matrix is used. In this approach, each video frame $X_{i}$ is divided into non-overlapping blocks of a size B×B. Then, each block is acquired by utilizing the Gaussian random projection measurement matrix $\Omega_{B}$ of a size $M_{B}\times B^{2}$. As a result, the measurement (or sensing) matrix for each frame is equal to

(4) $$\Phi_{i}=\left[\begin{array}{cccc} \Omega_{B} & 0 & 0 & 0\\ 0 & \Omega_{B} & 0 & 0\\ 0 & 0 & \ddots & 0\\ 0 & 0 & 0 & \Omega_{B} \end{array}\right].$$

First, the block-based sensing approach allows calculating the measurements separately for each block (i.e., it can be performed in parallel). Second, for the same reason, the recovery process can be performed in parallel as well. However, this approach has the following disadvantages:

1. As was shown in [23], the block-based measurements provide a much lower reconstruction quality compared with the global measurements which are acquired for a whole frame.
2. From an implementation point of view, the block-based sensing $y_{i}=\Phi_{i}x_{i}$ requires matrix multiplication with floating-point coefficients (i.e., it is not efficient from the encoding computational complexity point of view [28]).

In order to overcome the disadvantages mentioned above, in [23], we introduced the following global measurement (or sensing) matrix:

(5) $$\Phi_{i}=\left[\begin{array}{c} W\\ N_{i}-N_{i}W^{T}W \end{array}\right],$$

where W is a downsampling matrix of the *L*-level Haar transform, which is needed to compute low-resolution images of a size $\frac{W}{2^{L}}\times \frac{H}{2^{L}}$, and $N_{i}$ is $M_{i}-\frac{W}{2^{L}}\times \frac{H}{2^{L}}$ random rows of an orthogonal real-valued dragon noiselet transform (DNT) matrix [29]. The operation $y_{i}=N_{i}x_{i}$ with DNT is presented in Algorithm 1.
**Algorithm 1**: Forward (inverse) DNT**Input: **x1:$c\leftarrow N^{2}-1$2:**for **$j=0,\cdots ,\frac{N^{2}}{2}$**do**3:   k←j⊕c4:   $y_{j}\leftarrow x_{j}+x_{k}$5:   $y_{k}\leftarrow x_{j}-x_{k}$6:**end for **7:**for **$d=\left\lfloor \frac{c}{2}\right\rfloor ,\left\lfloor \frac{c}{4}\right\rfloor \cdots ,1$**do**8:   **for** $j=0,\cdots ,\frac{N^{2}}{2}$ **do**9:     k←j⊕c⊕d10:     $t\leftarrow y_{j}$11:     $y_{j}\leftarrow y_{j}-y_{k}$12:     $y_{k}\leftarrow t+y_{k}$13:   **end for**14:**end for**

First, the DNT can be applied only for a 1D vector of a power of two samples. Therefore, in [16], we proposed extending the input vector by $2^{N}-WH$ additional zero samples, where $N=\left\lceil \log_{2}\left(WH\right)\right\rceil$. Second, after forward and inverse DNT, each sample should be divided by $\sqrt{2^{N}}$. In order to avoid square root calculation and division, in [16], we proposed combining this operation with quantization in the following way. At the encoder side, we compute the quantized measurement as follows:

(6) $$y_{i}^{q}=\left\lfloor \frac{y_{i}}{2^{N-\left\lfloor N/2\right\rfloor +q}}\right\rfloor ,$$

where $Q=2^{q}$ is the quantization step, and at the decoder side, we dequantize the measurement as follows:

(7) $${\hat{y}}_{i}=\left\lfloor \frac{y_{i}^{q}}{2^{\left\lfloor N/2\right\rfloor -q}}\right\rfloor .$$

One can see that the DNT combined with quantization is performed for integer numbers and does not require division and multiplication. Since the downsampling matrix of the *L*-level Haar transform can be also performed without division and multiplication, the considered measurements matrix in Equation (5) is very attractive for low-complexity encoding.

### 2.3. Measurement Matrix Transmission

According to the CS framework, after DNT, we need to take $M_{i}-\frac{W}{2^{L}}\times \frac{H}{2^{L}}$ out of $2^{N}$ coefficients located at pseudo-random positions. Here, from the reconstruction quality point of view, these positions should be different for the neighboring frames. In [16], we proposed generating only 8 different pseudo-random vectors of a size of 10, corresponding to sensing rates $R_{i}=\left\{3\%,5\%,10\%,15\%,20\%\right\}$, where each vector element defines a distance between two coefficients to be included in the output bit stream. For example, for $R_{i}=10\%$, the following vectors are used:

(8) $$\begin{array}{c} v_{0}=\left\{19,9,11,17,12,6,7,5,12,2\right\},\\ v_{1}=\left\{5,16,9,2,6,2,15,14,12,19\right\},\\ v_{2}=\left\{2,19,4,18,14,16,13,1,2,11\right\},\\ v_{3}=\left\{5,17,7,18,7,18,7,4,12,5\right\},\\ v_{4}=\left\{14,17,4,8,16,10,2,8,18,3\right\},\\ v_{5}=\left\{20,5,4,17,5,2,20,9,11,7\right\},\\ v_{6}=\left\{12,18,6,4,15,7,8,6,7,17\right\},\\ v_{7}=\left\{13,4,11,5,7,20,4,16,9,11\right\}. \end{array}$$

If we use vector $v_{0}$, then the quantized measurements $y_{1}^{q},y_{20}^{q},y_{29}^{q},y_{40}^{q},\cdots$ will be included in the output bit stream. Here, both the encoder and the decoder use vector $v_{i\;\mathrm{mod}\;8}$ for the frame with an index *i*.

### 2.4. Measurements of Compression and Packetization

**Figure 1:**
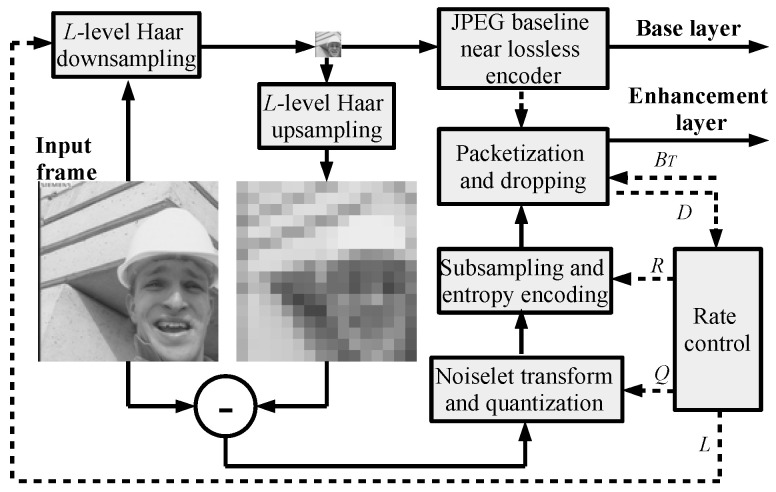
CS-JPEG intra-frame encoder.

The measurement matrix in Equation (5) produces two types of measurements which are compressed as in [16] (see Figure 1).

The low-resolution image is compressed by a JPEG baseline with a quality factor of 100 (near lossless mode). The quantized and subsampled DNT coefficients are binarized and compressed via a context-adaptive binary range coder (CABRC) [30], which is up to 40% faster than conventional adaptive binary arithmetic coders with bit renormalization [31]. Here, one binary context is used for the sign of a DNT coefficient, while its amplitude is unarily binarized and compressed using 64 binary contexts. The compressed DNT coefficients are packetized so that each packet also includes the frame index, the packet index, the sensing rate index, an absolute position of the first DNT coefficient within the packet, and other information needed for decoding. Moreover, CABRC is reinitialized for each new packet so that each packet can be decoded separately and used for reconstruction. All packets with DNT coefficients are embedded into an application part of the JPEG header. As a result, any JPEG-compatible software can decode the low-resolution part of a frame. The CS-JPEG decoder is used in order to find the full frame resolution. Let us also note that any other potential image coding, such as JPEG2000, H.264/AVC (Intra), or H.265/HEVC (Intra), can be used for low-resolution image compression. CS-JPEG uses JPEG due to its low complexity and simplicity.

### 2.5. The Soft Thresholding Operator Selection

In [16], we proposed using randomized VBM3D from [23] as a soft thresholding operator soft(.) in the CS-JPEG decoder. VBM3D originally was developed as a video denoising filter. However, as was shown in [32], such denoising filters can be considered as thresholding operators as well. VBM3D assumes that a video sequence has temporal similarity between blocks in different frames and similarity between blocks at different spatial locations within the same frame. Under this assumption, it performs denoising in two stages. At the first stage, for each reference block, several of the most similar blocks $b_{1},\cdots ,b_{K}$ in the current frame and neighbor frames are grouped into a 3D block. Then, the hard thresholding operator $hard(\left\{\left[b_{1};\cdots ;b_{K}\right],\Psi ,\sigma \right)$ is applied:

1. A sparsifying transform with a matrix Ψ is applied for a 3D block as $\theta =\Psi [b_{1};\cdots ;b_{K}]$.
2. The hard threshold transform coefficients $\tilde{\theta }=\left\{{\tilde{\theta }}_{i}\right\}$ are calculated as follows:
  (9) $$\tilde{\theta_{i}}=\left\{\begin{array}{c} 0,\;\mathrm{if}\left|\theta_{i}\right|<\sigma ,\\ \theta_{i},\;\mathrm{otherwise}. \end{array}\right.$$
3. A hard threshold block is calculated as $\left[{\hat{b}}_{1};\cdots ;{\hat{b}}_{K}\right]=\Psi^{-1}\tilde{\theta }$.

Since each pixel can be estimated by the hard thresholding of different 3D blocks, operator hard() also computes a weight coefficient $\gamma_{H}$ for all pixels estimates corresponding to the 3D block as $\gamma_{H}=\frac{1}{\sigma^{2}{∥\tilde{\theta }∥}_{0}}$, where ${∥\tilde{\theta }∥}_{0}$ is $l_{0}$-norm (number of non-zero coefficients) of $\tilde{\theta }$. Thereafter, the final estimate of each pixel is calulated as a weighted sum of the all estimates.

Video frames obtained at the first stage are used at the second stage as estimates of the noise-free frames in order to find similar blocks $b_{1},\cdots ,b_{K}$ more precisely. Then, instead of the hard thesholding, an empirical Wiener shrinkage operator $wiener(\left\{\left[b_{1};\cdots ;b_{K}\right],\Psi ,\sigma \right)$ is performed:

1. A sparsifying transform with matrix Ψ is applied for a 3D block as $\theta =\Psi [b_{1};\cdots ;b_{K}]$.
2. A shrinkage coefficients $\tilde{\theta }=\left\{{\tilde{\theta }}_{i}\right\}$ are calculated as $\tilde{\theta_{i}}=\theta_{i}\mu_{i}$, where $\mu_{i}=\frac{\theta_{i}^{2}}{\theta_{i}^{2}+\sigma^{2}}$.
3. A resulting block is calculated as $\left[{\hat{b}}_{1};\cdots ;{\hat{b}}_{K}\right]=\Psi^{-1}\tilde{\theta }$.

Here, the final estimate of each pixel is calculated as a weighted sum of all the estimates as well, where a weight coefficient for each estimate is computed as $\gamma_{W}=\frac{1}{\sigma^{2}{∥\mu ∥}_{2}^{2}}$.

As was shown in [16], the resulting CS-JPEG video codec with ISTA based on VBM3D significantly outperformed the other codecs based on CS, such as DISCOS [17], DCVS [18], MH-BCS [19], and MC-BCS [20]. However, the computational complexity of the VBM3D is very high. Therefore, in [21], we proposed a low-complexity ISTA based on simple spatial transforms (4×4, 8×8, and 16×16 DCT) and temporal transforms (1-D DCT of sizes 4, 8, and 16) which were randomly selected at each iteration, and we showed that it reduced the recovery time by around 300 times compared with the one with VBM3D. However, the low-complexity ISTA did not take into account the motion between neighbor frames. Therefore, its reconstruction performance was much worse than that of ISTA with VBM3D. As a result, a reconstruction algorithm combining a high recovery performance and low computational complexity should be developed.

### 2.6. Parameter Selection for the CS-JPEG Codec

There are three parameters in the CS-JPEG encoder: the number of levels for the Haar transform *L*, sensing rate *R*, and quantization step *Q* for DNT coefficients. At the decoder side, there are only two parameters: an initial threshold value $\sigma_{0}$ and the number of iterations *K*. In general, the problem of the codec parameter selection can be formulated as follows. For a given bit rate, we need to choose a triple (L,R,Q) at the encoder and a corresponding pair $\left(\sigma_{0},K\right)$ at the decoder which provide the maximum reconstruction quality. This task could be solved by a brute force algorithm which includes encoding and decoding with all possible combinations of the parameters. However, such an approach requires too many computations. Therefore, in [21], we proposed a heuristic separate optimization within three stages. At the first stage, we set the parameters $L,\sigma_{0}$, and *K* and performed encoding and decoding for all pairs (R,Q), where R∈{3%,5%,10%,15%,20%} and $Q\in \{2^{0},2^{1},\cdots ,2^{4}\}$. Then, for the selected pairs (R,Q), we obtained the best L∈{2,3,4,5}, shown in Table 1.

Finally, for the selected triples (L,R,Q), we performed the reconstruction with $\sigma_{0}\in \left\{10,\cdots ,140\right\}$ and K∈{50,100,200,500} and found that $\sigma_{0}=20$ and K=500 provided the best rate distortion results on average. However, the problem of parameter selection for a given target bit rate was not covered in [21], (i.e., a rate control algorithm should be developed).

## 3. Proposed Rate Control for the CS-JPEG Encoder

**Figure 2:**
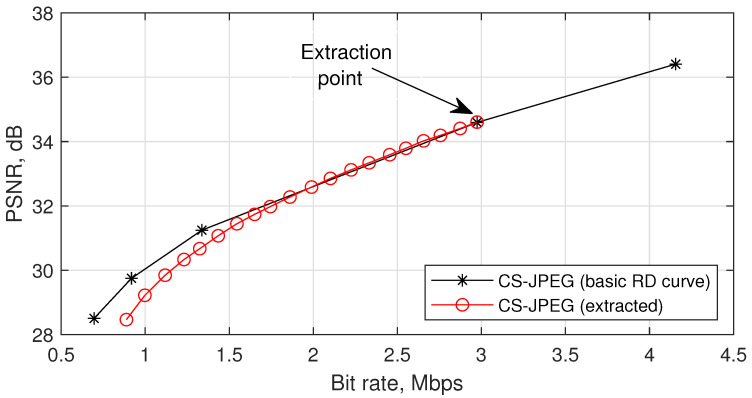
Illustration of the codec scalability for `Foreman’.

According to Table 1, the CS-JPEG encoder supports five basic sets of coding parameters {R,Q,L}. As a result, five basic RD points are provided. Since the measurements in different packets are compressed independently and assumed to have equal contributions to the decoded quality, the remaining RD points could be achieved by dropping the corresponding number of packets before transmission or storage. Figure 2 illustrates this idea. First, we built the basic RD curve. Second, we chose a bit stream corresponding to a sensing rate R=15%, removed 5% of the packets from it, and performed the decoding. Then, we removed packets from the resulting bit stream, and so on. One can see that the proposed codec provides a high level of quality scalability (i.e., a wide range of bit rates can be achieved by extraction or dropping the corresponding number of packets from the initial bit stream).

**Figure 3:**
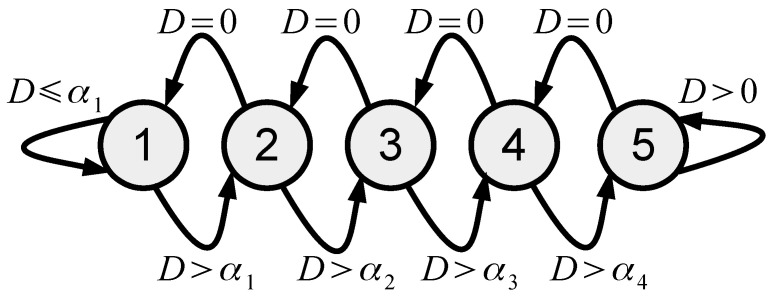
The proposed rate control state machine.

In this paper we propose using quality scalability in order to introduce the following rate control algorithm in the form of a state machine with five states $s_{i}\in \left\{1,2,\cdots ,5\right\}$, presented at Figure 3.

Each state $s_{i}$ defines the coding parameters for frame *i* as shown in Table 1. Let us define $B_{T}$ as the target number of bits for each frame and $B_{i}$ as the number of bits after compression of frame *i*, with the encoding parameters defined by the state $s_{i}$. The encoder uses the packet dropping mechanism, which allows it to store or transmit $\min\{B_{i},B_{T}\}$ bits and compute the frame dropping level as

(10) $$D_{i}=\frac{\max\{0,B_{i}-B_{T}\}}{B_{i}}.$$

The dropping level $D_{i}=0$ indicates that the encoder should generate more bits; in other words, we have

(11) $$s_{i+1}=\min\left\{1,s_{i}-1\right\}.$$

Otherwise, $D_{i}>0$ indicates that the encoder generates $B_{T}$ bits. However, if $D_{i}$ is too high, then the encoder wastes a lot of computational resources in order to acquire and compress a lot of measurements which are dropped. Moreover, from the RD performance point of view, it could be more efficient to use a lower sensing rate with different *Q* and *L* values (see Figure 2). Therefore, if

(12) $$D_{i}>\alpha \left(s_{i}\right),$$

where $\alpha \left(s_{i}\right)\approx \frac{B(s_{i}+1)}{B(s_{i})}\approx \frac{R(s_{i}+1)}{R(s_{i})}$, then the lower sensing rate is selected; in other words, we have

(13) $$s_{i+1}=\max\left\{5,s_{i}+1\right\}.$$

It could be noticed that the proposed rate control algorithm has relatively low computational complexity, since it computes only the dropping level $D_{i}$, which is compared with zero and $\alpha (s_{i})$ in order to define the triple {R,Q,L} for the next frame.

## 4. Proposed Fast Randomized Thresholding ISTA in the CS-JPEG Decoder

### 4.1. Basic Soft Thresholding for the ISTA

Let us introduce the following basic soft thresholding, presented in Algorithm 2. First, for a current reference block R of a size N×N with the left-top corner coordinates (i,j) in frame ${\tilde{X}}_{f}$, we apply hierarchical motion estimation (HME) [33]. HME returns the most similar blocks $B_{1},\cdots ,B_{F}$ with the corresponding motion vectors $v_{1},\cdots ,v_{F}$ such that block $B_{i}$ belongs to frame ${\tilde{X}}_{i}$. Second, these blocks are grouped into a 3D block of a size F×N×N, and then the 3D discrete cosine transform (DCT) coefficients $\theta =\{\theta_{i}\}$ for it are calculated. Then, empirical Wiener shrinkage [22] is performed for each coefficient as $\tilde{\theta_{i}}=\theta_{i}\mu_{i}$, where $\mu_{i}=\frac{\theta_{i}^{2}}{\theta_{i}^{2}+\sigma^{2}}$, a weight coefficient γ for the 3D block is calculated as $\gamma =\frac{1}{\sigma^{2}{∥\mu ∥}_{2}^{2}}$, and then inverse 3D DCT for the coefficients $\tilde{\theta }=\left\{{\tilde{\theta }}_{i}\right\}$ is performed in order to obtain the thresholded blocks ${\hat{B}}_{1},\cdots ,{\hat{B}}_{F}$. Third, the operator insert() creates two zero frames S and E and inserts a block ${\hat{B}}_{n}$ into frame S and a block of the same size containing only ones into frame E according to motion vector $v_{n}$. Finally, the sum of the weighted pixel values and the corresponding sum of weights are accumulated in buffers $X_{1},\cdots ,X_{F}$ and $W_{1},\cdots ,W_{F}$, respectively, and the resulting output frame is computed as ${\hat{X}}_{f}=X_{f}/W_{f}$, where the division is performed in an element-by-element manner.
**Algorithm 2**: Basic soft thresholding operator (*Version 1*)**Input: **${\tilde{X}}_{1},\cdots ,{\tilde{X}}_{F},\sigma$1:$X_{1}\leftarrow 0,\cdots ,X_{F}\leftarrow 0$, $W_{1}\leftarrow 0,\cdots ,W_{F}\leftarrow 0$2:**for **f=1,⋯,F**do**3:   **for** i=1,⋯,W−N **do**4:     **for** j=1,⋯,H−N **do**5:        $R\leftarrow getblock({\tilde{X}}_{f},i,j,N)$6:        $\{B_{1},v_{1},\cdots ,B_{F},v_{F}\}\leftarrow$HME$\left(i,j,R,{\tilde{X}}_{1},\cdots ,{\tilde{X}}_{F}\right)$7:        $\left\{\left[{\hat{B}}_{1};\cdots ;{\hat{B}}_{F}\right],\gamma \right\}\leftarrow wiener\left(\left[B_{1};\cdots ;B_{F}\right],\sigma \right)$8:        **for** n=1,⋯,F **do**9:          $\left\{S,E\right\}\leftarrow insert\left({\hat{B}}_{n},v_{n}\right)$10:          $X_{n}\leftarrow X_{n}+\gamma S$11:          $W_{n}\leftarrow W_{n}+\gamma E$12:        **end for**13:     **end for**14:   **end for**15:**end for**16:**for **f=1,⋯,F**do**17:   ${\hat{X}}_{f}\leftarrow X_{f}/W_{f}$18:**end for**

In contrast to VBM3D, Algorithm 2 does not have the first stage, where the frames ${\tilde{X}}_{1},\cdots ,{\tilde{X}}_{F}$ are hard thresholded in order to obtain better estimations for the motion estimation in the second stage. In our case, we assume that the frame estimations at iteration k−1 are good enough to use for motion estimation at iteration *k*. Moreover, in the motion estimation, we are not searching for the most similar blocks within the same frame. Both of these approaches allow reducing the computational complexity. Table 2 shows an average recovery time for F=16 and N=16, where the basic thresholding is called *Version 1*. One can see that the complexity is still too high and should be reduced.

### 4.2. Proposed Modifications of the Basic Soft Thresholding with 16×16×16 DCT

**Figure 4:**
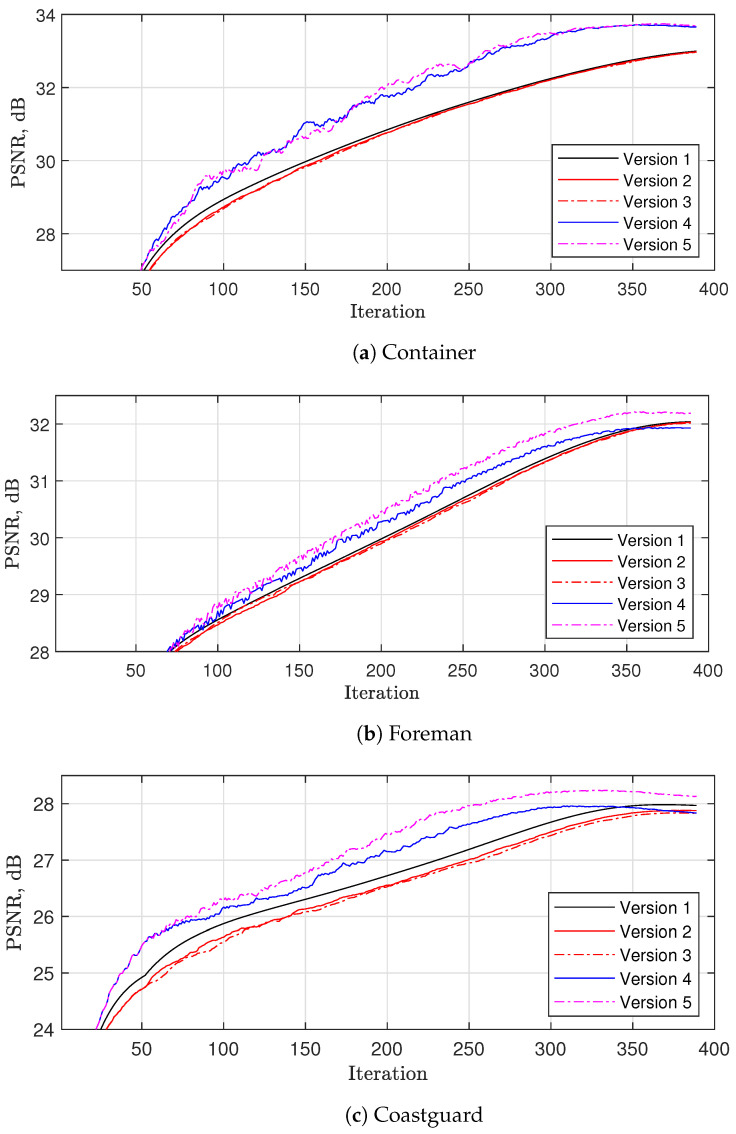
Comparison of different versions of thresholding with 16×16×16 DCT for R=5%. Here, *Version 1* is the thresholdig according to Algorithm 2, *Version 2* is *Version 1* with random selection of shifts $\left(s_{i},s_{j}\right)$ from set {0,N/2}, *Version 3* is *Version 2*, in which hierarchical motion estimation (HME) is used once per 30 iterations, while for the remaining iterations, only motion vector refinement via fast gradient motion vector searching is used, *Version 4* is *Version 3*, in which any motion estimation is performed at each iteration with a probability of 1/3 while zero-motion vectors are used with a probability of 2/3, and *Version 5* is *Version 4*, in which motion searching within the same frame is enabled.

In this paper, we propose the following four modifications of the basic soft thresholding:

1. Let us approximate the basic thresholding results by reducing the number of considered reference blocks. In the original VBM3D [22] only, we have
  (14) (i,j)∈{1,1+N/2+s,1+N+s,⋯},
  where s=0 is used, while in [23], *s* is randomly selected from a set s∈{0,1} at each iteration. Both approaches consider a significantly lower number of reference blocks, while the second one uses four times more combinations, which allows obtaining better performance. However, both of these approaches consider a limited number of different reference blocks used during the whole recovery process. Therefore, in *Version 2*, we propose using
  (15) $$\left\{\begin{array}{c} i\in \{s_{i},N+s_{i},2N+s_{i},\cdots \}\\ j\in \{s_{j},N+s_{j},2N+s_{j},\cdots \}, \end{array}\right.$$
  where both $s_{i}$ and $s_{j}$ are randomly selected from a set $s_{i},s_{j}\in \left\{0,N/2\right\}$ at each iteration. From one side, in contrast to [22,23], this approach uses a lower number of reference blocks at each iteration. As a result, it is more than 11 times faster than basic thresholding (see Table 2) and 3–5 times faster than VBM3D (see Table 3). From the other side, if *K* is high enough, this allows us to use almost all combinations of the reference blocks during the whole recovery process, providing the closest approximation to *Version 1* (see Figure 4).
2. As in [23], *Version 2* performs HME at each iteration, which is computationally expensive. Therefore, in *Version 3*, we assume that the estimated frames do not change significantly from iteration to iteration. Therefore, we can, for instance, perform HME only once per 30 iterations, while for the remaining iterations, we apply only the motion vector refinement performing fast gradient motion vector searching [33], utilizing the motion vectors estimated at the previous iteration as the initial vectors. As a result, the overall complexity is reduced by around four times compared with *Version 2*, keeping the same reconstruction performance (see Figure 4).
3. A further reduction in complexity can be achieved by avoiding motion estimation at all for some iterations (i.e., when zero motion vectors are used). In *Version 4*, we propose disabling motion estimation for the first $K_{0}=\left\lfloor 0.1K\right\rfloor$ iterations, while for $k>K_{0}$, the motion estimation is performed with a probability of 1/3. This allows reducing the thresholding complexity by 1.75 times compared with *Version 4* (see Table 2). Figure 4 shows that this approach has minor PSNR degradation for video sequences with fast and medium motion (see `*Foreman*’ and `*Coastguard*’) and noticeable PSNR improvement when the motion level is low (see `*Container*’). This can be explained in the following way. At initial iterations, the current estimates ${\tilde{X}}_{1},\cdots ,{\tilde{X}}_{F}$ look noisy such that the motion estimation is not able to obtain zero-motion vectors even for completely static videos. In contrast, the proposed solution allows using zero-motion vectors, which improves the reconstruction quality compared with both *Version 3* and VBM3D.
4. Since some videos have a high level of non-local similarities between the blocks within a frame, following VBM3D in *Version 5*, we propose collecting *F* blocks from *F* frames, which have the maximum similarity, so that more than one block for a given frame can be selected in the thresholding. This allows us to achieve a noticeable improvement in PSNR but with almost double the complexity compared with *Version 4*.

### 4.3. Proposed Thresholding Based on 3D DCT with Randomized Block Sizes

**Figure 5:**
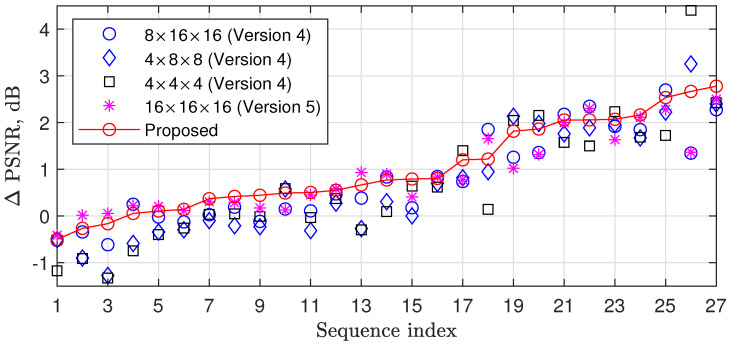
PSNR improvements related to [21] for the thresholding with 3D DCT block sizes 8×16×16, 4×8×8, 4×4×4 and 16×16×16 and the proposed thresholding in which the block size is selected from a set {8×16×16,4×8×8,4×4×4, and 16×16×16} at each iteration with a probability of 1/4.

The sparsity level, achieved through 3D DCT for a block of size of F×N×N, depends on the video properties. From the spatial sparsity point of view, a larger *N* is better for a flat area, while the smaller ones are better for areas with many details. From the temporal sparsity point of view, smaller *N* and *F* values allow finding more similar blocks in the neighbor frames when the motion level is medium or high, while for a low-motion area, a larger *F* value is better. Figure 5 shows PSNR improvements related to the recovery algorithm from [21] for different 3D DCT block sizes in case of compression of the first 16 frames of the test video sequences with a sensing rate R=5%.

As could be expected, different block sizes provide different results for different videos. In this paper, we propose exploiting the advantages of different transform sizes in the following way. At each iteration, we randomly select the transform size using equal probabilities. This approach does not increase the complexity. At the same time, Figure 5 shows that it provides an average improvement in PSNR from 0.13 to 0.36 dB compared with a case when one fixed transform size is used for all iterations and up to 3 dB compared with [21].

**Figure 6:**
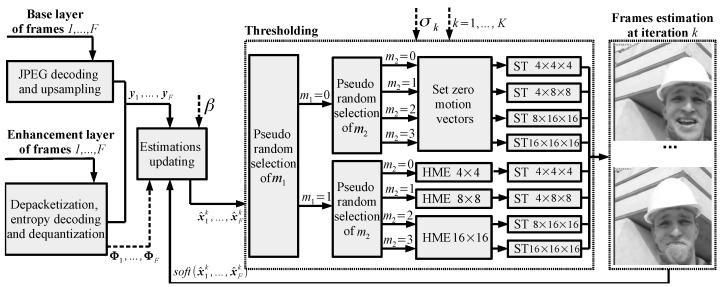
Proposed fast randomized ISTA for CS-JPEG decoder.

Following the reasoning described above, Figure 6 shows the proposed fast randomized ISTA as a part of the CS-JPEG decoder. At each iteration $k>K_{0}$, we first randomly select the mode $m_{1}\in \left\{0,1\right\}$ with a distribution $\left\{\frac{1}{3},\frac{2}{3}\right\}$. If $m_{1}=1$, then we randomly select mode $m_{2}\in \left\{0,1,2,3\right\}$ with a distribution $\left\{\frac{1}{4},\frac{1}{4},\frac{1}{4},\frac{1}{4}\right\}$. If $m_{2}=0$, then we perform motion estimation for the 4×4 blocks and the thresholding with 4×4×4 DCT. For $m_{2}=1,2,3$, we perform motion estimation for the 8×8, 16×16, and 16×16 blocks, respectively, and the thresholding with 4×8×8 DCT, 8×16×16 DCT, and 16×16×16 DCT, respectively. If $m_{1}=0$ or $k\leq K_{0}$, then we disable the motion estimation, set all the motion vectors to zero, and perform the thresholding.

**Figure 7:**
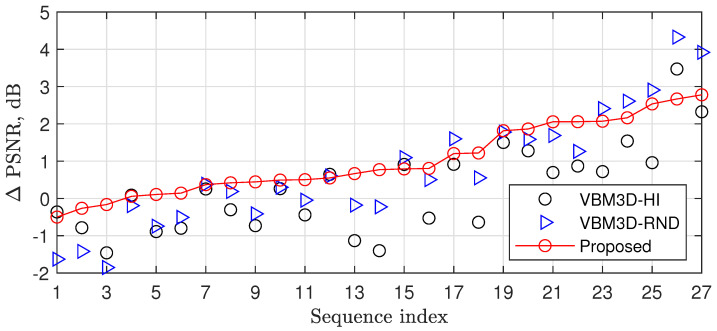
PSNR improvements related to [21] for thresholding using VBM3D-HI, VBM3D-RND, and the proposed randomized thresholding.

Figure 7 and Table 3 show a comparison of the proposed fast randomized ISTA and the ISTA with the original VBM3D [22] in a high profile (VBM3D-HI) and VBM3D from [23] with random selection of parameters as well (VBM3D-RND). One can see that, on average, the proposed ISTA outperformed VBM3D-HI and VBM3D-RND by 0.76 dB and 0.26 dB, having 20 and 32 times lower complexity, respectively.

## 5. Performance Evaluation

### 5.1. Test Video Sequences

The performance evaluation was obtained for 27 test video sequences available in [34]. All the sequences had a frame resolution of 352×288. In all cases, only the luminance (*Y*) component was considered. The test sequences were selected so that they had very different motion levels. In order to measure this numerically, let us introduce the following temporal similarity level *S*:

(16) $$\begin{array}{c} S=10\log_{10}\left(\frac{x_{max}^{2}}{d_{MC}}\right), \end{array}$$

where $x_{max}=255$ is the maximum possible value of the pixels in original video sequence and

(17) $$d_{MC}=\frac{1}{W\cdot H\cdot (F-1)}\sum_{f=2}^{F}\sum_{i=1}^{W}\sum_{j=1}^{H}{\left(X\left(i,j,f\right)-X_{MC}\left(i,j,f-1\right)\right)}^{2},$$

where X(i,j,f) and $X_{MC}\left(i,j,f-1\right)$ are the values of the pixels with coordinates (i,j) in the original frame with a number *f* and the motion-compensated frame f−1 relative to frame *f*, respectively.

Table 4 shows the considered video sequences with the corresponding number of frames and the temporal similarity level *S*. Here, the sequences `*Bridge-close*’, `*Container*’, and `*Bridge-far*’ had the highest *S* values (i.e., the neighboring frames were almost the same), while `*Football*’, `*Mobile*’, and `*Flower*’ had very low similarity due to heavy motion of the camera and the objects.

### 5.2. Rate Control Precision Comparison

**Figure 8:**
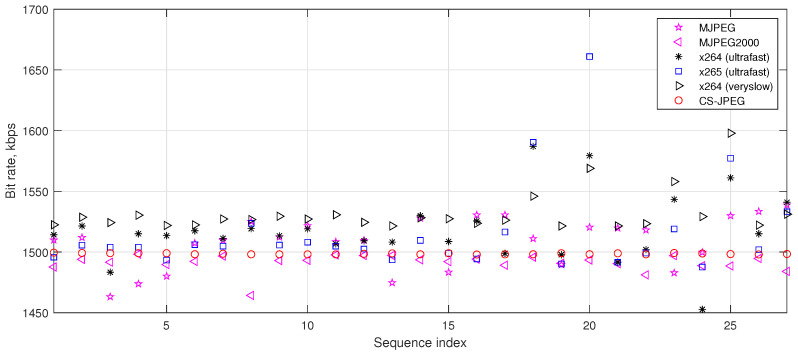
Bit rate provided by the considered codecs for $R_{T}=1500$ kbps.

In this paper, we compare the proposed rate control for the CS-JPEG codec (The CS-JPEG codec can be found at https://github.com/eabelyaev/csjpeg, accessed on 3 December 2022) with the corresponding algorithms in the most famous intra-frame encoders such as MJPEG [35] (MATLAB implementation), Motion JPEG2000 (MJPEG2000) [36], x264 [7], and x265 [24], which are fast software implementations of H.264/AVC [8] and H.265/HEVC [25] encoders, respectively. All the codecs were run without any software optimization tools, such as assembler optimization or threads. Here, *ultrafast* means that the x264 or x265 encoder is used in its fastest preset, while *veryslow* corresponds to the full RD optimization. Since MJPEG does not contain any rate control, we implement the following rate control based on a virtual buffer. For frame *i*, we select the JPEG quality factor $Q_{i}\in \left\{0,1,\cdots ,100\right\}$ as follows:

(18) $$Q_{i}=100-\left\lfloor \frac{100\cdot V_{i}}{V_{max}}\right\rfloor ,$$

where $V_{max}=fps\cdot D\cdot C$ is the size of the virtual buffer, fps is the frame rate, D=1 is the buffering delay, $C=\frac{R_{T}}{fps}$ is the virtual channel rate, and $V_{i}$ is the virtual buffer fullness after encoding frame i−1, which is calculated as follows:

(19) $$V_{i}=V_{i-1}+B\left(Q_{i-1}\right)-C,$$

where $B(Q_{i-1})$ is the number of bits after compression of frame i−1 with a quality factor $Q_{i-1}$. The rate control (Equation (18)) selects $Q_{i}$, depending on the virtual buffer fullness $V_{i}$, so that a decrease in $V_{i}$ leads to an increase in the quality factor $Q_{i}$ and vice versa. As a result, a bit rate close to the target one $R_{T}$ is provided. Similar rate controls are used in both x264 and x265. However, as can be seen in Figure 8, in many cases, the virtual buffer cannot guarantee a bit rate close enough to the target value $R_{T}$. MJPEG, x264, and x265 can provide a bit rate which is up to 2.6%, 5.8%, and 10.7% higher than $R_{T}$, respectively. This can be very critical for some video streaming applications. In contrast, both MJPEG2000 and the proposed rate control for CS-JPEG provided almost exact $R_{T}$ for all the considered test video sequences. Here, the proposed rate control had a higher precision than that in MJPEG2000.

### 5.3. Encoding Speed Comparison

Table 5 shows an encoding speed comparison for the considered codecs, measured in terms of the number of frames which could be compressed in one second by a 2.8 GHz CPU. Let us notice that both x264 and x265 were developed and optimized for many years by professional software engineers, while CS-JPEG was implemented in C-language by the author of the paper, who is not an expert in software development. Nevertheless, one can see that, on average, the CS-JPEG encoder was 2.2, 39.8, 1.9, 26.2, and 30.5 times faster than MJPEG, MJPEG2000, *x264-ultrafast*, *x264-veryslow*, and *x265-ultrafast*, respectively. These results practically prove that video coding based on CS has a much lower encoding complexity than the traditional block-based or wavelet-based intra codecs (i.e., it could be attractive for video applications having significant limitations in computational resources or the battery lifetime of an upstreaming device).

### 5.4. Rate Distortion Comparison via the PSNR Metric

Let us compare the rate distortion performance of the considered codecs using the peak signal-to-noise ratio (PSNR) as an objective visual quality metric. The PSNR is defined as

(20) $$\begin{array}{c} PSNR=10\log_{10}\left(\frac{x_{max}^{2}}{d}\right), \end{array}$$

where

(21) $$d=\frac{1}{W\cdot H\cdot F}\sum_{f=1}^{F}\sum_{i=1}^{W}\sum_{j=1}^{H}{\left(X\left(i,j,f\right)-\hat{X}\left(i,j,f\right)\right)}^{2},$$

where X(i,j,f) and $\hat{X}\left(i,j,f\right)$ are the values of the pixels with coordinates (i,j) in the original and reconstructed frames with a number *f*, respectively.

**Figure 9:**
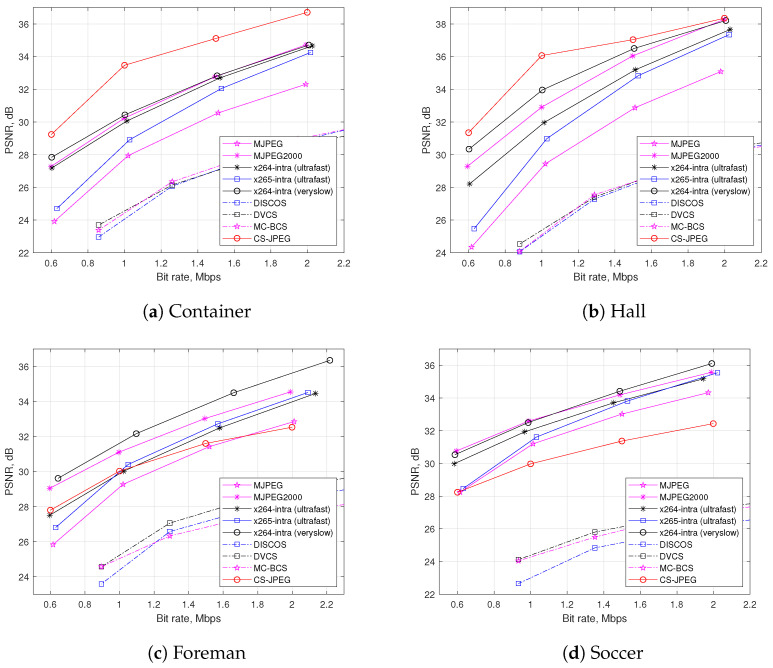
Rate distortion comparison using PSNR metric.

Figure 9 shows a rate distortion performance comparison for the test video sequences `*Container*’, `*Hall*’, `*Foreman*’, and `*Soccer*’. One can see that for `*Container*’ and `*Hall*’, the proposed CS-JPEG codec outperformed all the block-based codecs, while for `*Foreman*’ and `*Soccer*’, it provided similar or even worse results. This could be explained in the following way. The recovery performance of CS-JPEG highly depends on the statistical properties of a video sequence. On one hand, CS-JPEG exploits the temporal similarity between neighboring frames (i.e., higher similarity means better reconstruction). On the other hand, if the temporal similarity is low, then the traditional block-based intra codecs could provide better performance, since they exploit the spatial similarity of the pixels within a frame more efficiently.

**Figure 10:**
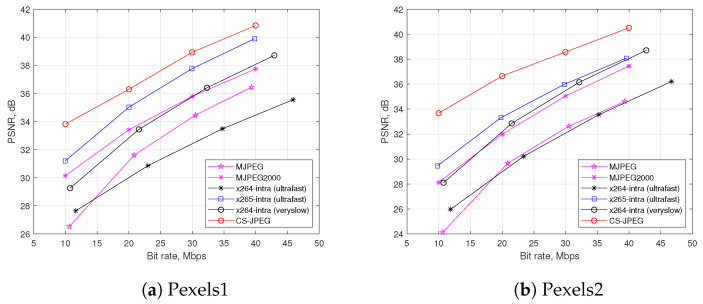
Rate distortion comparison using PSNR metric for Full HD videos.

As a reference, Figure 9 also shows the rate distortion performance for other codecs based on CS such as DISCOS, DCVS, and MC-BCS. One can see that the proposed CS-JPEG provided significantly better results. Therefore, we further compared CS-JPEG only with the block-based codecs, such as x264, x265, MJPEG, and wavelet-based MJPEG2000. Finally, x265-intra (ultrafast) provided surprisingly worse compression performance than x264-ultrafast. This could be explained by H.265/HEVC originally being oriented for video sequences with higher frame resolutions. In order to demonstrate this, Figure 10 presents comparisons for two test video sequences [21] with a frame resolution of 1920×1088. For such a resolution, x265-intra (ultrafast) provided much better performance than both x264-intra (ultrafast) and x264-intra (veryslow).

Table 6 shows the BD-PSNR [37] provided by the CS-JPEG codec in comparison with the intra codecs mentioned above. Here, the positive values mean that CS-JPEG provided better performance. One can see that if the temporal similarity level S>30 dB, then in the most of the cases, CS-JPEG outperformed the competitors, including *x264-veryslow* and MJPEG2000. If S<30 dB, then the temporal similarity was too low, so the traditional block-based or wavelet-based intra-coding had more advantages. Using this observation, we can conclude that CS-JPEG is more attractive for applications with low and medium motion levels between neighboring frames.

### 5.5. Rate Distortion Comparison via the SSIM Metric

For objective visual quality evaluation, we also used the Structural Similarity Index (SSIM) [38], which measures the similarity between two images and ranges from 0 to 1. Table 7 shows the BD-SSIM provided by the CS-JPEG codec in comparison with the intra codecs. One can see that, as in the previous case, CS-JPEG provided better performance for S>30 dB.

### 5.6. Comparison via Flickering Level

The objective PSNR and SSIM metrics are calculated for only a single frame. Therefore, these metrics are not always good enough for quality assessment of a video sequence. Let us also evaluate inter-frame flickering, which is a commonly seen video coding artifact. It is perceived mainly because of the increased inter-frame difference between neighboring frames after compression compared with the corresponding difference in the original video. As a result, flicker is more easily seen in static areas. Table 8 shows the flickering level [39] for the considered codecs. One can see that for a similarity level S>26.5 dB, the proposed CS-JPEG codec provided a much lower flickering level (except for `*Bridge-far*’) compared with the other block-based codecs, while in some cases, wavelet-based MJPEG2000 had the lowest flickering level.

### 5.7. Subjective Comparison

**Figure 11:**
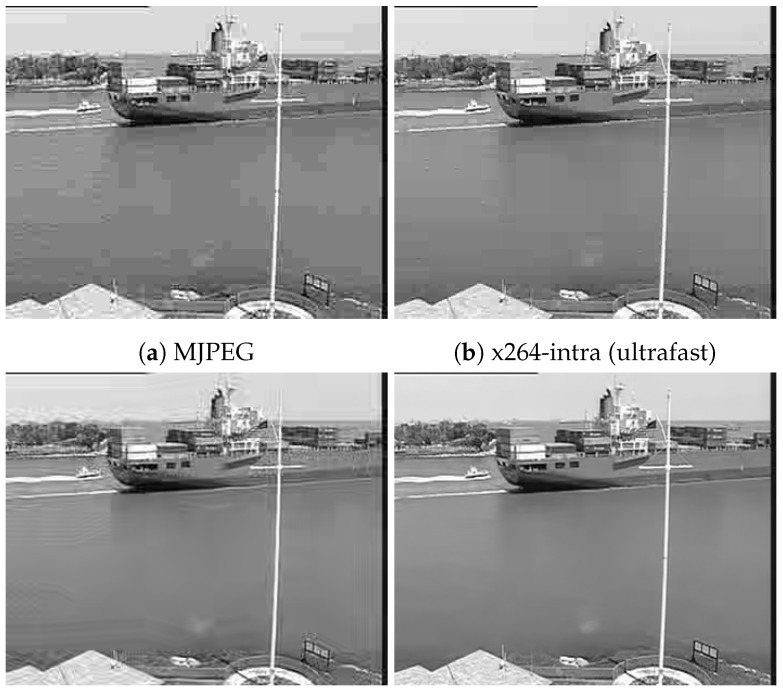
Frame with index 150 at $R_{T}=600$ kbps for `Container’.

**Figure 12:**
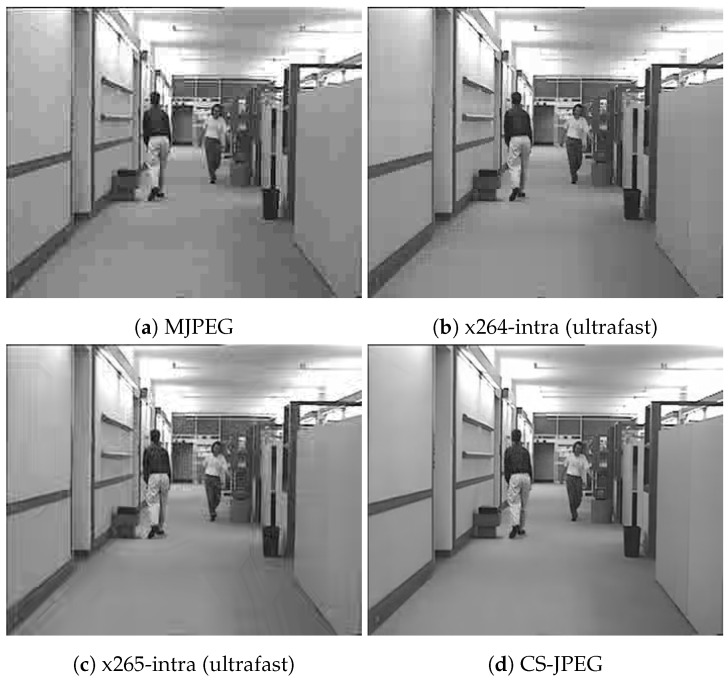
Frame with index 150 at $R_{T}=600$ kbps for `Hall’.

**Figure 13:**
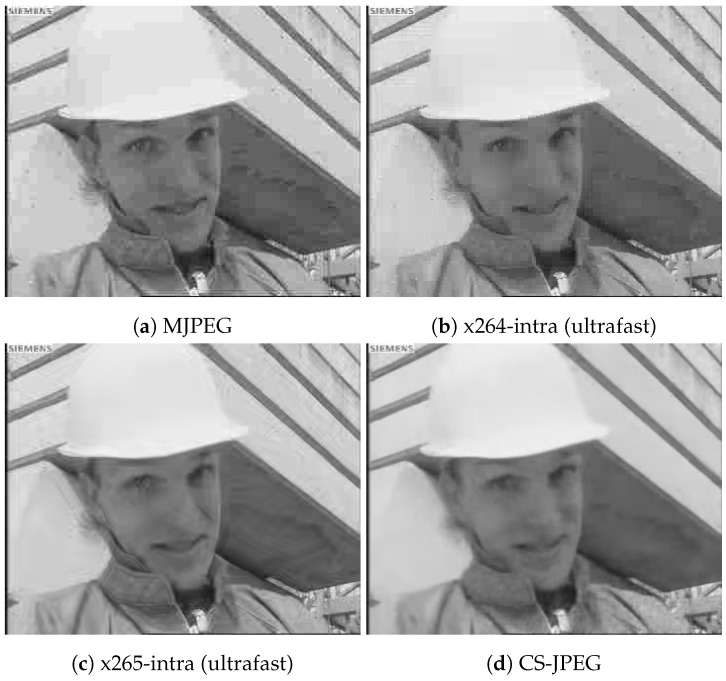
Frame with index 150 at $R_{T}=600$ kbps for `Foreman’.

**Figure 14:**
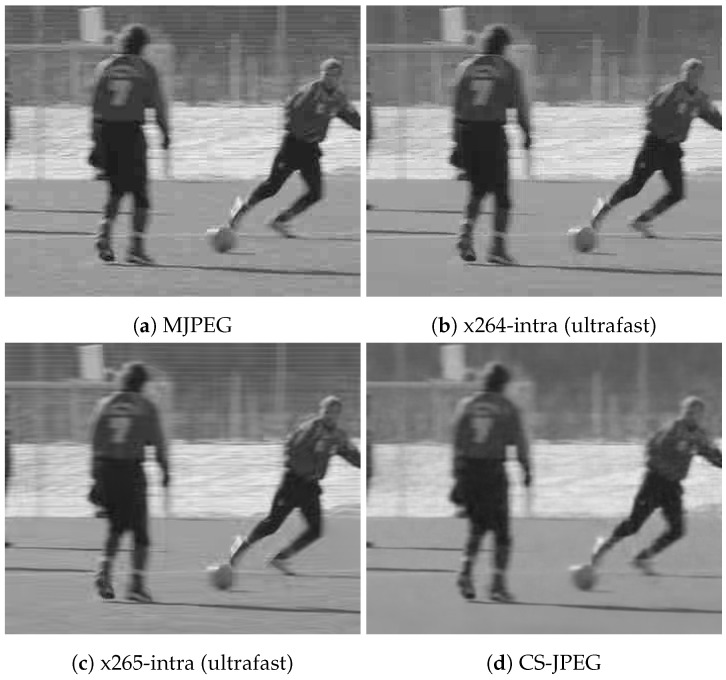
Frame with index 150 at $R_{T}=600$ kbps for `Soccer’.

Figure 11, Figure 12, Figure 13 and Figure 14 show frame 150 for `*Container*’, `*Hall*’, `*Foreman*’, and `*Soccer*’ at a target bit rate $R_{T}=600$ kbps. It could be noticed that MJPEG, x264, and x265 produced very strong blocking artifacts, while CS-JPEG was free of them.

## 6. Conclusions

In this paper we summarized the CS-JPEG codec with intra-frame encoding and inter-frame decoding inspired by compressive sensing. We demonstrated that when compared with the traditional block-based intra codecs, it is significantly less complex and provides a higher level of scalability. Moreover, for video sequences with low and medium motion levels, it also provided better rate distortion performance. This makes CS-JPEG more attractive for video applications having significant limitations in computational resources or battery lifetime for an upstreaming device.

## Figures and Tables

**Table 1:** CS-JPEG coding parameters.

State $s_{i}$	$L(s_{i})$	$R(s_{i})$	$Q(s_{i})$	$\alpha (s_{i})$
1	2	20%	2	0.4
2	3	15%	2	0.4
3	3	10%	2	0.6
4	4	5%	4	0.5
5	4	3%	4	–

**Table 2:** Recovery time for frame of size 352×288 for thresholding with 16×16×16 DCT on 2.8 GHz CPU.

Version	1	2	3	4	5
Frame recovery time (s)	1390.7	119.5	29.7	17.0	33.6

**Table 3:** An average frame recovery time for VBM3D and the proposed algorithm on a 2.8 GHz CPU.

Recovery Algorithm	VBM3D-HI	VBM3D-RND	Proposed
Frame recovery time (s)	303.6	498.0	15.2

**Table 4:** Test video sequences.

Test Video	Number of Frames	Temporal Similarity *S* (dB)
Bridge-close	300	59.7
Container	300	48.6
Bridge-far	300	45.2
Akiyo	300	39.9
Mother-daughter	300	37.2
Students	300	37.2
Hall	300	36.2
Waterfall	260	36.1
Silent	300	32.2
Deadline	300	32.0
News	300	31.5
Pamphlet	300	31.4
Bowing	300	30.6
Paris	300	30.3
Sign-irene	300	29.5
Tempete	260	26.8
Harbour	300	26.5
Carphone	300	26.4
Crew	300	25.5
Foreman	300	24.5
Coastguard	300	24.4
City	300	24.2
Ice	240	22.9
Soccer	300	21.1
Football	260	20.0
Mobile	300	19.5
Flower	250	17.7

**Table 5:** Encoding speed in frames per second for `Foreman’ with 352×288 resolution and 300 frames on 2.8 GHz CPU.

$R_{T}$ (kbps)	*MJPEG*	*MJPEG2000*	*x264-intra*		*x265-intra*	*CS-JPEG*
			*Ultrafast*	*Veryslow*	*Ultrafast*	
600	223	11.9	346.8	24.3	17.0	551.7
1000	221	12.0	271.3	19.1	15.6	516.8
1500	213	11.8	216.6	15.9	14.9	444.4
2000	199	11.8	176.9	13.8	14.4	380.9

**Table 6:** BD-PSNR provided by CS-JPEG compared with different intra-frame codecs.

*Test Video*	*S* (dB)	*MJPEG*	*MJPEG2000*	*x264-intra*		*x265-intra*
				*ultrafast*	*veryslow*	*ultrafast*
Bridge-close	59.7	3.54	1.13	1.58	1.31	2.67
Container	48.6	5.27	2.71	2.92	2.54	4.19
Bridge-far	45.2	1.83	−0.57	−0.78	−0.28	0.68
Akiyo	39.9	4.49	1.27	2.85	0.53	3.40
Mother-daughter	37.2	2.41	0.10	1.07	−0.87	1.02
Students	37.2	4.80	2.44	3.26	2.27	4.09
Hall	36.2	5.91	2.17	3.18	1.41	4.32
Waterfall	36.1	1.72	0.27	1.08	0.57	1.28
Silent	32.2	3.40	1.27	2.47	1.36	2.65
Deadline	32.0	6.46	3.55	4.15	2.69	5.24
News	31.5	6.26	3.35	4.28	2.35	5.59
Pamphlet	31.4	6.60	3.48	4.60	2.59	5.11
Bowing	30.6	5.27	1.75	2.79	0.71	3.65
Paris	30.3	5.11	2.37	3.29	1.89	4.07
Sign-irene	29.5	0.92	−1.78	−1.25	−2.44	−0.19
Tempete	26.8	0.57	−1.13	0.01	−1.03	0.01
Harbour	26.5	2.01	0.15	1.26	0.05	0.96
Carphone	26.4	1.38	−2.03	−0.48	−2.40	−0.18
Crew	25.5	−1.47	−3.36	−2.45	−3.63	−2.06
Foreman	24.5	0.77	−1.30	−0.18	−1.97	−0.28
Coastguard	24.4	0.69	−1.27	−0.52	−1.25	−0.52
City	24.2	−0.08	−1.38	−0.86	−1.34	−0.36
Ice	22.9	−0.40	−3.19	−2.23	−4.05	−0.15
Soccer	21.1	−1.18	−2.74	−2.22	−2.79	−1.66
Football	20.0	−2.23	−4.24	−3.35	−4.35	−2.66
Mobile	19.5	−0.49	−1.72	−1.24	−1.88	−0.44
Flower	17.7	−0.62	−2.28	−1.86	−2.28	−1.15
***Average***	**31.0**	**2.33**	**−0.04**	**0.79**	**−0.38**	**1.45**

**Table 7:** BD-SSIM provided by CS-JPEG compared with different intra-frame codecs.

*Test Video*	*S* (dB)	*MJPEG*	*MJPEG2000*	*x264-intra*		*x265-intra*
				*Ultrafast*	*Veryslow*	*Ultrafast*
Bridge-close	59.7	0.08	0.04	0.06	0.02	0.08
Container	48.6	0.07	0.04	0.04	0.01	0.07
Bridge-far	45.2	0.02	−0.01	−0.00	−0.01	0.01
Akiyo	39.9	0.04	0.01	0.04	0.01	0.04
Mother-daughter	37.2	0.04	0.00	0.02	−0.01	0.02
Students	37.2	0.09	0.05	0.08	0.03	0.09
Hall	36.2	0.09	0.03	0.05	0.01	0.06
Waterfall	36.1	0.06	0.00	0.06	0.00	0.07
Silent	32.2	0.09	0.03	0.08	0.02	0.08
Deadline	32.0	0.13	0.06	0.08	0.02	0.12
News	31.5	0.08	0.04	0.06	0.01	0.09
Pamphlet	31.4	0.10	0.04	0.07	0.02	0.08
Bowing	30.6	0.06	0.01	0.03	0.00	0.04
Paris	30.3	0.14	0.06	0.10	0.03	0.13
Sign-irene	29.5	0.02	−0.01	0.00	−0.02	0.01
Tempete	26.8	0.01	−0.04	0.02	−0.06	0.02
Harbour	26.5	0.07	0.01	0.06	−0.02	0.05
Carphone	26.4	0.04	−0.02	0.02	−0.03	0.02
Crew	25.5	−0.03	−0.07	−0.04	−0.08	−0.04
Foreman	24.5	0.02	−0.03	0.02	−0.05	0.01
Coastguard	24.4	−0.01	−0.06	−0.02	−0.10	−0.04
City	24.2	−0.02	−0.07	−0.03	−0.08	−0.02
Ice	22.9	0.01	−0.01	−0.00	−0.02	0.02
Soccer	21.1	−0.04	−0.08	−0.05	−0.10	−0.05
Football	20.0	−0.09	−0.13	−0.09	−0.16	−0.08
Mobile	19.5	−0.05	−0.11	−0.07	−0.14	0.00
Flower	17.7	−0.05	−0.10	−0.08	−0.12	−0.04
***Average***	**31.0**	**0.036**	**−0.013**	**0.018**	**−0.030**	**0.031**

**Table 8:** Flickering level at $R_{T}=600$ kbps.

*Test Video*	*S* (dB)	*CS-JPEG*	*MJPEG*	*MJPEG2000*	*x264-intra*		*x265-intra*
					*Ultrafast*	*Veryslow*	*Ultrafast*
Bridge-close	59.7	2.0	3.5	**1.6**	2.1	3.9	2.9
Container	48.6	**1.1**	5.7	1.8	2.3	4.7	4.2
Bridge-far	45.2	1.9	0.8	**0.7**	0.7	1.2	1.2
Akiyo	39.9	**0.4**	5.1	1.2	1.5	2.2	2.5
Mother-daughter	37.2	**1.2**	2.3	**1.2**	1.4	1.9	2.3
Students	37.2	**0.8**	4.8	2.0	2.4	5.1	4.1
Hall	36.2	**1.9**	8.5	**1.9**	2.8	3.8	5.2
Waterfall	36.1	**2.3**	3.9	2.4	2.8	5.9	3.6
Silent	32.2	**1.0**	1.8	2.0	2.3	4.9	4.1
Deadline	32.0	**1.2**	4.3	2.7	3.0	7.4	5.5
News	31.5	**0.7**	2.8	1.8	2.4	4.3	4.3
Pamphlet	31.4	**0.9**	4.5	2.0	2.3	4.3	4.0
Bowing	30.6	**0.9**	3.8	1.3	1.5	2.4	2.7
Paris	30.3	**1.4**	1.7	3.0	4.0	10.3	8.1
Sign-irene	29.5	1.5	3.8	**1.3**	1.5	1.9	2.4
Tempete	26.8	4.0	4.2	**3.5**	4.7	8.0	6.2
Harbour	26.5	5.1	4.6	**3.8**	5.2	9.8	7.6
Carphone	26.4	2.8	5.2	**2.1**	2.7	2.7	3.8
Crew	25.5	4.3	3.4	**1.8**	2.1	2.3	2.6
Foreman	24.5	3.5	5.1	**2.1**	2.7	2.9	3.6
Coastguard	24.4	7.2	5.5	**2.0**	2.3	4.3	3.2
City	24.2	7.0	4.8	**2.4**	2.8	4.0	3.1
Ice	22.9	**0.9**	3.2	1.7	1.7	1.4	2.7
Soccer	21.1	5.3	2.7	1.3	**1.3**	1.9	1.7
Football	20.0	5.6	2.8	1.6	**1.3**	2.7	1.6
Mobile	19.5	11.1	**4.7**	5.6	6.6	9.7	8.8
Flower	17.7	9.0	1.5	1.8	**1.4**	2.3	2.2

## Data Availability

The CS-JPEG codec as well as additional visual comparisons can be found at https://github.com/eabelyaev/csjpeg, accessed on 3 December 2022.
